# Literary Notes

**Published:** 1895-11

**Authors:** 


### Literary Notes.

The *Archives of Pediatrics* will commence its thirteenth year with the January, 1896, number, under the business management of E. B. Treat, publisher, of New York. The *Archives* has been for twelve years the only journal in the English language devoted exclusively to diseases of children, and has always maintained a high standard of excellence.

The new management proposes several important changes in its make-up, increasing the text 15 per cent. and enlarging its scope in every way. This will give room for the fuller contributions of additional collaborators who have been secured for the various departments.

The editorial management will remain in the hands of Floyd M. Crandall, M. D., adjunct professor of pediatrics, New York Polyclinic, and chairman of section on pediatrics, New York Academy of Medicine.

---

Mr. W. B. Saunders, Philadelphia, announces an American Year-Book of Medicine, edited by Dr. George M. Gould, of Philadelphia, that will be ready for delivery January 1, 1896. It is Mr. Saunders' intention to publish this work yearly in the expectation that it will have an extensive sale throughout the United States as well as abroad. It is the special purpose of the editor to review contributions to American journals as well as those of Europe, thus making the year-book international in character.

The work will be replete with original and selected illustrations skilfully reproduced, for the most part in Mr. Saunders' own studios, established for the purpose, thus insuring accuracy in delineation, affording efficient aids to a right comprehension of the text and adding to the attractiveness of the volume.

---

Weight chart for infants is a new chart published by the Just's Food Company, by which the weight of an infant, from birth to two years of age, may be recorded from time to time. Such a record is not only valuable to the physician, showing at a glance whether the child is properly gaining in weight, but it will also be an interesting souvenir to the mother. On the chart is a model line as a guide for an average child. By referring to our advertising pages it will be seen that this chart will be sent free to any address. It is certainly something novel, and we believe will prove of great value.

---

Physicians' Protective Association.—At a well-attended meeting of the Buffalo Physicians' Protective Association, held at the Academy of Medicine parlors, Wednesday afternoon, October 16, 1895, the following officers were elected for the ensuing year: President, Dr. John N. Goltra; vice-president, Dr. Thomas F. Dwyer; recording secretary, Dr. John G. Meidenbauer; financial secretary, Dr. J. N. Kraus; treasurer, Dr. William G. Ring.

